# Undefilled blood tube containing EDTA: Is it an inappropriate sample for HbA1c assay?

**DOI:** 10.11613/BM.2023.010901

**Published:** 2023-02-15

**Authors:** Berrak Guven, Ismail Benice, Murat Can

**Affiliations:** Department of Biochemistry, Faculty of Medicine, Zonguldak Bülent Ecevit University, Zonguldak, Turkey

**Keywords:** HbA1c, blood sample collection, preanalytical error, diabetes mellitus

## Abstract

**Introduction:**

Blood samples having inappropriate volume are a substantial part of preanalytical errors. Inadequate sample volume for glycated haemoglobin (HbA1c) test may be a common problem of patients with diabetes mellitus having vascular changes. In this study, we compared HbA1c concentrations of underfilled and appropriately filled blood collection tubes.

**Materials and methods:**

To compare HbA1c concentrations, blood samples were collected into 2 mL tubes containing K3-EDTA from 109 subjects. Two blood samples (underfilled and appropriately filled) were drawn from a patient by the same personnel and materials. HbA1c measurements were assayed on a Cobas 6000 analyser module c 501 (Roche Diagnostics, Mannheim, Germany). The HbA1c% results were compared by t-test and Wilcoxon’s signed-rank statistical methods (SPSS Inc., Chicago, USA). Bias analysis was performed using Microsoft Excel 4.0.

**Results:**

Underfilled samples were classified three groups (group 1, N = 44; group 2, N = 36; and group 3, N = 29) according to the filling ratio of the samples; 0.5 mL and below (< 25%), 0.5-1.0 mL (25-50%), and 1.0-2.0 mL (> 50%), respectively. When we compared underfilled tubes with pairing filled tubes, there was a statistically significant difference only with tubes filled less than 25% (P = 0.030). Furthermore, we have done bias analysis between paired tubes according to the diagnostic cut-off value of 6.5%. The bias was more prominent in up to 50% underfilled blood tubes (1.1%), when HbA1c concentrations were below the diagnostic cut-off of 6.5%.

**Conclusions:**

We suggest that the blood tubes with EDTA for HbA1c measurement should be filled with at least 50% to avoid clinical variations.

## Introduction

Glycated haemoglobin (HbA1c) reflects the mean glycaemic level in the 120-day lifespan of the red blood cell. The American Diabetes Association recommends HbA1c, as a marker of diabetes mellitus (DM) diagnosis and control (*1*). The sampling of HbA1c has the advantages of being one blood sample and not requiring the patient to fast, over the glucose tolerance test, which is used as a diagnostic test for DM. According to the Clinical and Laboratory Standards Institute (CLSI), recommended samples for HbA1c measurement are ethylenediaminetetraacetic acid (EDTA) or heparin whole blood (*2*).

Unsuitable specimens for laboratory testing are the most important source of all laboratory errors (*3*). Especially, blood samples having insufficient or inappropriate volume form a substantial part (10-20%) (*4*). Partially underfilled blood tube is a common problem in patients with poor venous access or small veins (*5*, *6*). Accordingly, inadequate sampling may be possible in patients who had HbA1c test orders, due to the vascular changes in DM (*7*). To our knowledge, there is no comparison study in the literature on how underfilled samples affect HbA1c results. Our objective for this study was to compare HbA1c concentrations of underfilled and appropriately filled blood collection tubes.

## Material and methods

This study was performed during the period of one week in March 2022 at the Clinical Biochemistry Laboratory of Zonguldak Bülent Ecevit University Hospital. Written informed consent was obtained from all volunteers. The study was approved by the clinical research ethics committee of Zonguldak Bülent Ecevit University Faculty of Medicine (Approval Number: 23.02.2022/2022/04).

### Samples

Blood specimens were taken from 109 adult patients who were routinely ordered for HbA1c tests. Venous blood samples were drawn according to the recommendations of the CLSI, Document GP41 by the same personnel and materials. The samples were collected in 2 mL K3-EDTA blood collection tubes (Becton, Dickinson and Company, Franklin Lakes, USA). Two blood collection tubes with EDTA from each patient at the same time were taken, one of them was for routine HbA1c measurement, which was filled to its capacity, while another tube was underfilled. Transportation and analysis of both tubes were at the same time.

### HbA1c assay procedure

Glycated haemoglobin was measured using an immunoturbidimetric assay (Tina-quant HbA1c Gen. 3) on the Cobas 6000 analyser (Roche Diagnostics, Mannheim, Germany). The assay is certified by the National Glycohemoglobin Standardization Program (NGSP) and standardized by the International Federation of Clinical Chemistry and Laboratory Medicine (IFCC). The measurements were performed according to the manufacturer’s instructions using the same lots of reagents, calibrators, and control samples. Quality control evaluations were performed by two levels of control samples and all internal quality control results were within acceptable ranges in the study period. We have verified the analytical imprecision of the Cobas HbA1c assay using the CLSI EP5-A2 protocol (*8*). Within-run coefficients of variation (CVs) were 1.1% and 0.5%, and between-run CVs were 1.1% and 1.7%, for normal and high-level control, respectively. The HbA1c results were expressed in NGSP units as percentages (%) and calculated with the following equation from A1c/Hb rate: HbA1c (%) = (A1c/Hb) x 91.5 + 2.15.

### Statistical analysis

Statistical analyses were performed using SPSS statistical software version 18 (SPSS Inc., Chicago, USA). Normality of data distribution was assessed using the Shapiro-Wilk test. The results were presented as median, interquartile range and minumum-maximum values, because distribution was not normal. Wilcoxon matched pairs signed ranks test was used for comparison between underfilled and filled blood collection tubes. The level of significance for all statistical comparisons was set as P < 0.05. The biases between the results of % HbA1c were calculated as (% HbA1c underfilled) - (% HbA1c filled) using Microsoft Excel 4.0 and were presented with 95% confidence interval (95% CI).

## Results

We compared 109 underfilled whole blood samples with their appropriately filled pairs. Underfilled samples were divided into three groups according to the filling ratio of the samples: group 1 with the volume of 0.5 mL and below (< 25%) in 44 samples, group 2 with the volume between 0.5 and 1.0 mL (25-50%) in 36 samples, and group 3 with the volume between 1.0 and 2.0 mL (> 50%) in 29 samples. Comparisons of HbA1c concentrations between underfilled and filled samples are presented in Table 1. There was a significant difference for HbA1c% in tubes filled less than 25% (P = 0.030).

**Table 1:** Comparison of HbA1c concentrations in underfilled and appropriately filled tubes

**HbA1c (%)**	**Group 1** **(N = 44)**		**Group 2** **(N = 36)**		**Group 3** **(N = 29)**	
	**< 0.5 mL**	**2.0 mL**	**0.5-1.0 mL**	**2.0 mL**	**1.0-2.0 mL**	**2.0 mL**
Median (IQR)	6.2 (5.5-8.6)	6.1 (5.4-8.5)	6.5 (5.8-7.4)	6.4 (5.7-7.5)	6.6 (6.0-8.2)	6.5 (6.1-8.2)
Min-Max	5.0 - 12.0	4.9 - 11.7	4.9 - 10.9	4.9 - 11.1	5.2 - 10.1	5.3 - 10.0
P	0.030		0.150		0.810	
Group 1 – samples with filling volume 0.5 mL and below (< 25%). Group 2 - samples with filling volume between 0.5 and 1.0 mL (25-50%). Group 3 - samples with filling volume between 1.0 and 2.0 mL (> 50%). IQR- interquartile range. Min - lowest value. Max - highest value. Statistical significance was set at P < 0.05.						

To further determine the effect on the HbA1c concentration between underfilled and filled samples, we evaluated the HbA1c according to the diagnostic cut-off value of 6.5% (*9*). Of the 109 patient samples, 56 (51%) were below 6.5% and 53 (49%) were above 6.5%. To compare whether there was any significant difference between the paired samples, bias analysis was used. When evaluated according to the cut-off, as Table 2 shows, the bias is more prominent in patients having less than 6.5% in tubes filled with 50% or less.

**Table 2:** Biases obtained in tubes with underfilling ratios compared to standard volume according to the cut-off value for HbA1c

**HbA1c (%)**	**Filling ratio of tubes**		
	**< 25%**	**25-50%**	**> 50%**
Number of samples with HbA1c < 6.5%	25	17	14
Mean bias (95% Cl)	0.1 (- 0.2 to 0.3)	0.1 (- 0.2 to 0.3)	0 (- 0.2 to 0.3)
Mean bias (%)	1.3	1.1	- 0.5
Number of samples with HbA1c ≥ 6.5%	19	19	15
Mean bias (95% Cl)	0 (- 0.5 to 0.3)	0 (- 0.5 to 0.2)	0 (- 0.6 to 0.2)
Mean bias (%)	0	0	0.2
CI - confidence interval.			

## Discussion

Our study demonstrates that underfilling only up to 25% in EDTA-blood tubes may generate a statistically significant difference for HbA1c. When the comparisons were detailed by the bias analysis, for patients having < 6.5% HbA1c concentrations, we also found a positive bias in tubes filled 50% or less. According to our results, when HbA1c is used as a diagnostic marker in tubes filled with 50% or less, there is a possibility the clinical interpretation that may range from “normal” to “diabetic”. Small errors in HbA1c concentrations may have a high impact on the clinical decision, so bias should be as close to zero as possible.

The differences in our results can also be attributed to the analytical variability of the HbA1c assay. The overall performance of the HbA1c assay was evaluated by determining precision. The obtained CVs were in accordance with the manufacturers’ recommendation and in the acceptable range (*10*). In addition, the analytical variability does not appear to be a possible explanation as other tube volumes are not affected equally.

There are a limited number of analytes for which underfilling has been investigated in samples with anticoagulants (*11*-*13*). Generally, it is thought that up to 75% underfilling in tubes containing anticoagulant additives may rarely generate a clinically significant bias (*3*). Although there is no comparable study in the literature, it was reported by relatively few samples, that underfilled K2-EDTA or K3-EDTA blood collection tubes have no impact on HbA1c concentrations (*14*). Since the comparability of blood volume proportion in tubes with EDTA for HbA1c measurement has not yet been studied, our results may serve other laboratories that have problems with inadequate samples with a recommendation for at least 50% as the minimum sample volume.

The main limitation of our study is the unequal number of available samples in all groups. Second, analysis is limited to a specific type of instrument, the Cobas 6000, and its reagents.

In conclusion, we suggest that blood tubes with EDTA for HbA1c measurement should be filled with at least 50% to avoid clinical variations.
